# Elevated Random Luteinizing Hormone is an Unreliable Indicator for Pubertal Suppression in Girls Treated with Monthly Leuprolide for Idiopathic Central Precocious Puberty

**DOI:** 10.4274/jcrpe.galenos.2018.2018.0213

**Published:** 2019-09-03

**Authors:** Pattara Wiromrat, Ouyporn Panamonta

**Affiliations:** 1 Khon Kaen University Faculty of Medicine, Department of Pediatrics, Division of Endocrinology, Khon Kaen, Thailand

**Keywords:** Central precocious puberty, final adult height, leuprolide acetate, monitoring, pubertal progression, random luteinizing hormone

## Abstract

**Objective:**

Longitudinal data regarding random luteinizing hormone (LH) concentrations in patients with idiopathic central precocious puberty (ICPP) during treatment are limited. Therefore, we sought to evaluate random LH and estradiol concentrations during monthly leuprolide injection and their associations with pubertal progression and final adult height (FAH) in girls with ICPP.

**Methods:**

Medical records of 27 girls with ICPP who had attained FAH were reviewed. Patients’ height, weight, Tanner stage, growth rate (GR), bone age, random LH measured by both immunoradiometric and immunochemiluminescent methods, follicular-stimulating hormone (FSH) and estradiol levels were monitored until FAH.

**Results:**

Treatment was started at a mean (±standard deviation) age of 8.1±0.6 years with mean duration of 3.9±0.2 years. At six months of follow-up, random LH (p=0.048), FSH (p<0.001) and estradiol (p=0.023) concentrations were decreased compared with baseline. Thereafter, random LHs were well suppressed. GRs gradually decreased to prepubertal norm by month 12. Seventeen patients (63%) exhibited pubertal LH concentrations at least once during treatment visits. Furthermore, 43 of a total 116 (37%) LH measurements were found elevated. However, those patients with elevated random LH did not show signs of pubertal progression. After treatment, mean FAH was greater than predicted adult height (p<0.0001) and target height (p=0.03). At no time points of treatment did random LH, FSH and estradiol correlate with GRs or FAH.

**Conclusion:**

Elevated random LH is commonly found in ICPP girls during monthly leuprolide treatment. However, these elevations were not associated with clinical progression of puberty or decreased FAH, suggesting that it is not a reliable method for CPP monitoring.

**What is already known on this topic?**

To date, there has been no consensus method for biochemical monitoring for idiopathic central precocious puberty (ICPP). Previous studies have shown that girls with CPP commonly have pubertal luteinizing hormone (LH) on random measurement, particularly in those treated with long-acting gonadotropin-releasing hormone analog implant. However, data in girls treated with monthly leuprolide acetate are limited. Moreover, there has been no study examining random LH or its association with growth rates  and final adult height (FAH) in a longitudinal fashion.

**What this study adds?**

This study demonstrated that random LH was elevated in ~60% of ICPP girls treated with monthly leuprolide with a magnitude of rising LH higher than previously reported. In respect to fact that elevated random LH was not associated with pubertal progression, or decreased FAH, it does not appear to be a reliable method for ICPP monitoring.

## Introduction

Idiopathic central precocious puberty (ICPP) is one of the most common endocrine disorders in girls. Gonadotropin-releasing hormone analog (GnRHa) is the mainstay of therapy for pubertal suppression. This treatment also improves final adult height (FAH) (1,2) and reduces negative psychosocial consequences (3,4). However, the biochemical monitoring for ICPP treatment remains challenging since there are no robust data demonstrating universal cut-off levels of random or stimulated LH for defining pubertal suppression (1,5,6,7). Moreover, the association between a lack of biochemical suppression with clinical progression and its influence on growth rates (GR) or FAH have not been evaluated.

To date, suppressed stimulated LH has been widely used in several research projects to determine treatment efficacy in ICPP (5,8,9). Nevertheless, some hospitals, including ours, have employed random (basal/unstimulated) LH for treatment monitoring because this approach is less invasive, less time-consuming and more convenient for resource-limited settings (7,10). The limited number of short-term studies (10,11) have shown that random LH was commonly found elevated during 3-monthly and annual regimens for GnRHa treatment. However, these data are limited when using a monthly preparation of leuprolide treatment (8). Further, longitudinal data from this monitoring are needed to better understand the changes of random LH over time. Therefore, we sought to longitudinally assess the serum concentrations of random LH, follicular-stimulating hormone (FSH) and estradiol in patients receiving monthly leuprolide injection and to assess associations with pubertal progression and FAH in girls with ICPP.

## Methods

### Patients

This study protocol was approved by the Khon Kaen University Ethical Committee (approval number; HE591411). Since this is a retrospective study and our patient data were anonymised, informed consent was not required by our Ethical Committee. During the period between January 2002 and December 2016, 420 girls were referred for precocious puberty evaluation to our Pediatric Endocrine Clinic, Khon Kaen University Hospital.

Inclusion criteria were: 1) breast development (Tanner stage ≥2) under the age of 8 years; 2) advanced bone age (BA) ≥1 year; 3) GnRHa-stimulated LH ≥6.9 IU/L (12,13,14); 4) normal brain and pituitary magnetic resonance imaging; 5) receiving monthly GnRHa treatment.

There were 142 girls diagnosed as ICPP. One hundred and ten girls received monthly GnRHa treatment. Forty-seven girls reached FAH with complete medical records. Of these, 20 girls were excluded due to markedly advanced BA (≥13 years) at initial presentation, as this BA represents ~95% of FAH, and increased random LH concentrations may have less effect on the FAH compared to patients with lesser bone maturation. Therefore, there were 27 girls included in the analysis.

### Treatment

Treatment was started in patients with 1) rapid progression, 2) BA advancement ≥2 years, or 3) differences in predicted adult height (PAH) and target height (TH) of ≥1.5 standard deviation (SD) score at initial or during follow-up. All patients received a monthly 3.75 mg depot leuprolide acetate injection until the chronological age (CA) of 12 years. During treatment, no patient showed any clinical characteristics of pubertal progression (a change in breast Tanner stage, increased GR or vaginal bleeding), therefore, none received treatment modification.

### Follow up Examination and Hormonal Measurement

Height, weight and Tanner stage of breast and pubic hair were assessed every 3-6 months until the patients attained FAH. Height was measured to the nearest 0.1 cm using a stadiometer; and body weight to the nearest 0.1 kg. GR was calculated as a change in height during a 1-year interval. BA was assessed at the initial visit and before treatment discontinuation. Pre-treatment PAH was estimated using the accelerated table of the Bayley and Pinneau method (15). All clinical parameters were assessed by the same pediatric endocrinologist. FAH was defined as a GR ≤0.5 cm/year or a BA ≥16 years.

Morning blood samples for serum random (basal) LH, FSH and estradiol were collected at 7-8 am, before the regular scheduled leuprolide injection, every 3-6 months at the pediatric endocrine clinic in the first year of treatment, and then every 6 or 12 months if no clinical progression was evident. During 2002-2013, serum LH and FSH were measured using a sensitive IRMA (RIA-gnost^®^, monoclonal mouse antibodies, hLH and hFSH; CIS Bio International, Gif-Sur-Yvette, France) with a lower detection limit of 0.1 and 0.15 IU/L with inter-assay coefficient variations of 9.4% and 8.7%, respectively. After 2011, LH and FSH were analyzed using a more sensitive immunocemiluminescent assay (ICMA) from Esoterix Laboratories (Calabasas Hills, CA, USA) with a detection limit of 0.02 IU/L for both hormones and inter-assay coefficients of variation were 10.7% for LH and were 9.0% for FSH. In summary, 27/27, 25/25, 25/25, 17/22 and 9/17 random LH levels were analyzed using IRMA at 6, 12, 24, 36 and 48 months of treatment time points, respectively, and the rest were analyzed on ICMA. Estradiol levels were measured by an ICMA (Immulite 2000, Siemens Medical Solutions Diagnostics, Los Angeles, CA, USA) with a detection limit of 5 pg/mL. Hormonal values falling below the lower detection levels were taken as the lower detection levels. The random pubertal LH concentrations were defined as ≥0.6 IU/L for IRMA (12,13) and ≥0.3 IU/L for ICMA (10).

### Statistical Analysis

Statistical analysis was performed using SPSS v18 (IBM Inc., Chicago, Ill., USA). Data were presented as number, percentages, mean ± SD and median (Q1, Q3). A t-test, paired t-test or chi-square test were used to test the differences between groups as appropriate. Repeated measures ANOVA was performed to compare two repeated measurements in the same individuals. Correlations were evaluated using Spearman correlation coefficients. Non-parametric tests were used for non-normally distributed data. P value <0.05 was considered significant.

## Results

### Patient Characteristics

Baseline patient characteristics are shown in Table 1. Patients reported their mean ± SD age of breast onset at 6.9±0.1 years, however, treatment was started at mean age of 8.1±0.2 years with a mean duration of 3.9±0.2 years. The reason for a delay in treatment initiation was mainly due to lack of knowledge about their disease. All patients reported good treatment compliance, and none had missed or delayed the scheduled leuprolide injection. As expected, the patients had typical features of ICPP including breast development, tall stature, advanced BA and elevated random or stimulated LH concentrations.

### GRs and FAH

Mean GR±SD at pre-treatment and 6, 12, 24, 36 and 48 months during treatment were 8.5±2.5, 6±1.2, 5.6±1.1, 4.1±1.5, 4.1±1.3 and 2.6±1.4 cm/year, respectively. After treatment, mean FAH was greater than initial PAH (p<0.0001) and TH (p=0.03, Table 2).

### Serum LH, FSH and Estradiol Concentrations

**Figure 1:**
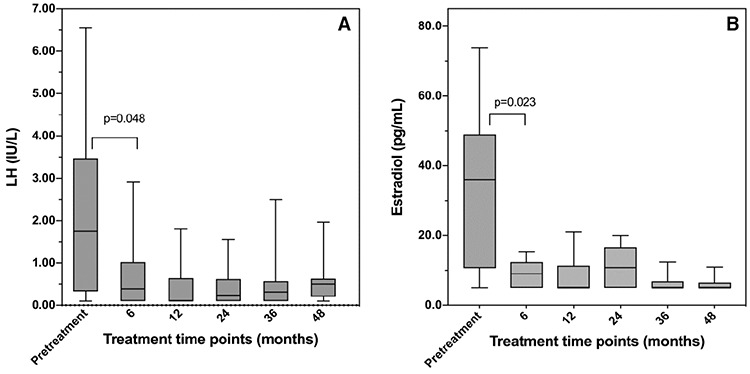
Random LH (A) and estradiol (B) concentrations before and during treatment. A) At month six, random LH concentrations were significantly lower than pre-treatment values (p=0.048). Thereafter, there were no statistical differences in random LH concentrations between each consecutive time point (6, 12, 24, 36 and 48). B) At month six, serum estradiol concentrations were significantly lower than pre-treatment values (p=0.023). There were no statistical differences in serum estradiol concentrations between each consecutive time point (month 6, 12, 24, 36 and 48). Data are presented as box and whisker plots. The center lines, lower edges and upper edges of the boxes indicate median, 25^th^- and 75^th^ percentiles, respectively. The whiskers indicate 5^th^-95^th^ percentile. LH: luteinizing hormone

Figure 1 shows random LH and estradiol concentrations during treatment. At six months of follow-up, the median serum LH [1.7 (0.3, 3.5) *vs* 0.46 (0.02, 1.14) IU/L; p=0.048], FSH [4.2 (2.6, 5.8) *vs* 1.6 (1.3, 2.1) IU/L; p<0.001) and estradiol [36 (11, 53) *vs* 9 (4, 12) pg/mL; p=0.023)] significantly declined from pre-treatment concentrations. Thereafter, the overall LH concentrations remained suppressed and tended to rise again at 36 and 48 months of follow-up. The median (Q1, Q3) random LH concentrations measured on IRMA were 0.4 (0.1, 1.02) at 6 months, 0.11 (0.1, 0.64) at 12 months, 0.24 (0.1, 0.62) at 24 months, 0.34 (0.07, 0.82) at 36 months and 0.6 (0.22, 0.85) at 48 months of treatment. The LH concentrations measured on ICMA at 36 and 48 months of treatment were 0.2 (0.09, 0.52) and 0.24 (0.23, 0.61), respectively. Median FSH values were 1.6 (0.82, 2.7), 1.8 (1.2, 2.6), 1.6 (1.2, 2.5), 1.4 (0.6, 1.7) and 0.6 (0.54, 1.7) at 12, 24, 36 and 48 months of treatment, respectively.

During treatment, 37% of a total of 116 LH measurements were within the pubertal range (≥0.6 IU/L for IRMA and ≥0.3 IU/L for ICMA). Among these, 10/27 (37%), 7/25 (30%), 7/25 (28%), 9/22 (41%) and 10/17 (61%) of the patients had elevated random LH at month six (range, 0.85-2.92 IU/L), month 12 (range, 0.6-1.8 IU/L), month 24 (range, 0.61-1.8 IU/L), month 36 (range: 0.30-0.60 IU/L for ICMA and 0.60-2.5 IU/L for IRMA) and month 48 (range: 0.3-0.83 for ICMA and 0.6-2 IU/L for IRMA), respectively. Moreover, 17 out of 27 individuals (63%) exhibited pubertal LH at least once at some point during treatment. However, these patients showed no signs of pubertal progression and thus none received treatment modification. Of a total of 112 estradiol measurements, 29% were above the lower detection limit (5 pg/mL). However, all values were in the prepubertal range of <20 pg/mL (16) throughout the treatment duration.

### Correlation

Random LH did not correlate at any of the time points with GR (Table 3) or FAH (data not shown). Also, there was no correlation between random LH, FSH, estradiol and body mass index (BMI) during treatment (data not shown). Neither FSH nor estradiol correlated with BMI, GRs and FAHs at any time points during treatment (data not shown).

### Patients with Frequently Elevated Random LH

Table 4 shows the clinical characteristics of three patients who presented with markedly advanced BA (BA-CA ~ 4.7-5.6 years), compromised PAH and frequent elevations in random LH during treatment. However, they did not show any signs of pubertal advancement. They each attained FAH which was higher than their PAH without any therapeutic modification.

## Discussion

Monitoring patients with ICPP is a clinical challenge since there is no consensus in method for monitoring. Failure  HPG-axis suppression can cause short FAH (1). In this study, we demonstrated longitudinal serum random LH concentrations during treatment and their relationship with pubertal progression and FAH.

Our study demonstrated that random LH was elevated in 37% of total measurements in ICPP patients during monthly leuprolide treatment with the magnitude of increase as high as 2.9 IU/L for IRMA and 0.62 IU/L for ICMA. Moreover, two-thirds of our patients exhibited pubertal LH levels at least once at some time points during follow-up and 30-60% of patients had random LH concentrations in the pubertal range during each year of treatment. Yet, none had pubertal progression by clinical examination. There are only three studies evaluating random LH concentrations or prevalence of increased random LH in CPP patients during GnRHa treatment as the main outcome [one in patients with trimonthly GnRHa injection (7) and the others in patients receiving  annual GnRHa implant (10,11)]. These studies showed similar proportions of patients with elevated random LH compared to our study results. Several findings in random LH concentrations in ICPP patients during various forms of GnRH treatment were reported (5,8,10,11,17). Neely et al (8) found that their subjects treated with a higher dose (7.5-15 mg) of monthly leuprolide had a satisfactory clinical and biochemical (random LH) suppression. Their patients’ maximal random LH concentrations in each follow-up visit were also lower than what was found in our patients (immunofluorometric assay, IFMA; 0.5-0.8 IU/L *vs* 1.8-2.9 IU/L). However, our patients were treated with a relatively lower dose of leuprolide (3.75 mg, 90-140 µg/kg) which is a commonly used dose in most Asian countries (18,19). Elevated random LH was also reported in 50-60% of ICPP patients treated with histrelin implant, without   pubertal progression (10,11). However, stimulated LH was well suppressed in these patients. Similar findings were also demonstrated in a study using monthly leuprolide (20) and another study using 3-monthly leuprolide (17). In contrast, Brito et al (5) reported that two out of 18 patients had pubertal breakthrough, although there was no specific report regarding “pubertal progression”, which was confirmed by a modest increase in random (0.9-1.1 IU/L, IFMA) and stimulated LH (4.3-5.7 IU/L). Therefore, these data indicated that elevated random LH is an unreliable marker for pubertal suppression.

Several studies have shown a correlation between random (basal) and stimulated LH concentrations, including Lee et al’s (7) study, which is the only work suggesting the random (basal) LH cut-off value. This study showed that basal LH (ICMA) ≥0.6 IU/L had an optimal sensitivity (80%) and  specificity (70%) for predicting stimulated LH levels of >4 IU/L. Again, this study did not demonstrate the relationship between increased basal LH or increased stimulated LH with clinical parameters, such as BA or clinical progression.

Our data also demonstrated that elevated random LH was not associated with GR nor FAH. There has been only one study (21) showing a similar result of lacking relationship between random LH and GR. That study also found that random LH had a negative association with the changes in PAH (r=-0.309; p<0.05), assessed by regular BA examination. To our knowledge, there have been no studies demonstrating the association between either random or stimulated LH and FAH. Our study showed that random LH was not correlated with FAH. Although two thirds of patients experienced pubertal LH, mean FAH was higher than PAH and at the upper range of TH. Further, the FAH and net height gain after GnRHa treatment in our patients with elevated random LH were similar to what have been previously reported in patients with suppressed stimulated LH (6,18,19,22,23). It is also worth mentioning that our three patients with frequently elevated random LH did not exhibit any signs of pubertal progression, and eventually attained FAH which was higher than their PAH and TH without treatment modification. Taken together, our results indicated that elevated random LH, in the context of no clinical progression being observed, may not have a negative effect on FAH.

In animal study, random LH was elevated during a continuous buserelin infusion, yet the pulsatile fashion was abolished (24,25) that resulted in ineffective sex hormone production. This finding may explain why our patients did not exhibit clinical signs of pubertal progression in response to increased random LH. Moreover, an alteration in the ratio of immunoreactive to bioactive gonadotropins (26) has also been described in patients receiving GnRHa. However, these hypotheses have never been tested in ICPP patients. Further research is needed.

In addition to random LH, we examined the association between estradiol concentration and pubertal advancement. We found that ~30% of the patients had slightly elevated estradiol concentrations above the lower detection limit (5-20 pg/mL) which are, however, considered prepubertal concentrations. In correlation analysis, we did not find associations between estradiol and random LH, BMI, GR nor FAH. These findings are incongruent with previous reports (21,27,28,29). Other studies also demonstrated that estradiol did not relate to BA maturation (21,25).

### Study Limitations

Our study has some weaknesses. First, we did not regularly assess BA, therefore, we could not evaluate the influence of increased random LH on skeletal maturation. Second, this is a retrospective study, in which we did not evaluate the stimulated LH concentrations at the time when elevated random LH was found. This led to an inability to assess the unknown effect of stimulated LH on GR and FAH. Our study also has a relatively small sample size. However, to our knowledge, this research is the first to demonstrate the longitudinal data of random LH concentrations throughout the treatment duration of ICPP, as well as the association with clinical markers of pubertal progression and FAH.

## Conclusion

In conclusion, we reported the longitudinal evidence that elevated random LH is commonly found in ICPP girls during monthly leuprolide treatment. However, the increase in random LH is not associated with clinical progression of puberty nor with FAH, suggesting that this method is unreliable and may not be useful for ICPP monitoring. More studies are needed to evaluate the relationship between elevated basal or stimulated LH and BA maturation or FAH.

## Figures and Tables

**Table 1:**
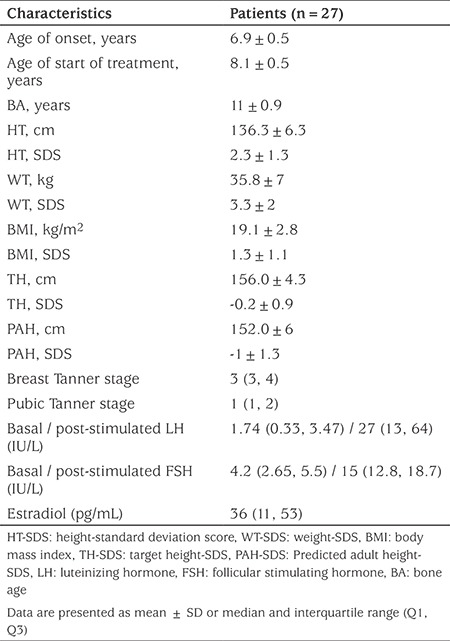
Baseline patient clinical and hormonal characteristics

**Table 2:**
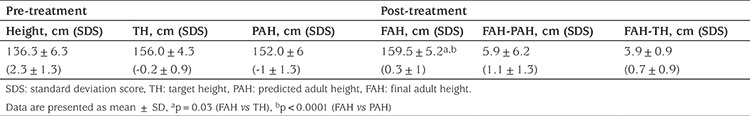
Final height outcome in 27 girls with idiopathic central precocious puberty

**Table 3:**
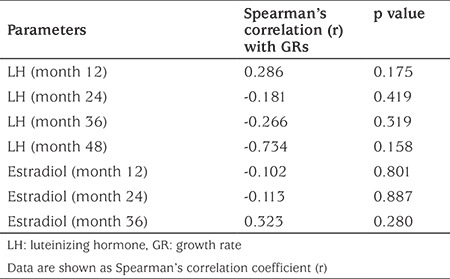
Correlation analysis between random luteinizing hormone and estradiol concentrations with growth rates at corresponding time points

**Table 4:**
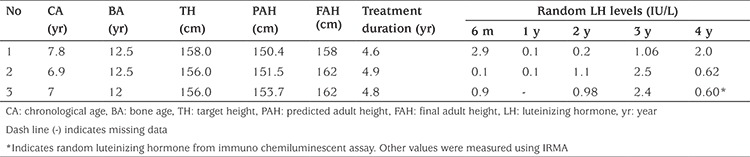
Clinical, hormonal characteristics and final adult height in three idiopathic central precocious puberty patients with persistent elevation of random luteinizing hormone levels during treatment
